# Circulating Endocannabinoids and Insulin Resistance in Patients with Obstructive Sleep Apnea

**DOI:** 10.1155/2016/9782031

**Published:** 2016-01-19

**Authors:** Xiaoya Wang, Qin Yu, Hongmei Yue, Jiabin Zhang, Shuang Zeng, Fenfen Cui

**Affiliations:** ^1^Department of Respiratory Medicine, The First Hospital of Lanzhou University, Lanzhou 730000, China; ^2^The First Clinical Medical College of Lanzhou University, Lanzhou 730000, China

## Abstract

*Objectives*. The purpose of this study is to investigate the relationship between plasma endocannabinoids and insulin resistance (IR) in patients with obstructive sleep apnea (OSA).* Methods*. A population of 64 with OSA and 24 control subjects was recruited. Body mass index (BMI), waist circumference, lipids, blood glucose and insulin, homeostasis model of assessment for insulin resistance index (HOMA-IR), anandamide (AEA), 1/2-arachidonoylglycerol (1/2-AG), and apnea-hypopnea index (AHI) were analyzed.* Results*. Fasting blood insulin (22.9 ± 7.8 mIU/L versus 18.5 ± 7.2 mIU/L, *P* < 0.05), HOMA-IR (2.9 ± 1.0 versus 2.4 ± 0.9, *P* < 0.01), AEA (3.2 ± 0.7 nmol/L versus 2.5 ± 0.6 nmol/L, *P* < 0.01), and 1/2-AG (40.8 ± 5.7 nmol/L versus 34.3 ± 7.7 nmol/L, *P* < 0.01) were higher in OSA group than those in control group. In OSA group, AEA, 1/2-AG, and HOMA-IR increase with the OSA severity. The correlation analysis showed significant positive correlation between HOMA-IR and AHI (*r* = 0.44, *P* < 0.01), AEA and AHI (*r* = 0.52, *P* < 0.01), AEA and HOMA-IR (*r* = 0.62, *P* < 0.01), and 1/2-AG and HOMA-IR (*r* = 0.33, *P* < 0.01). Further analysis showed that only AEA was significantly correlated with AHI and HOMA-IR after adjusting for confounding factors.* Conclusions*. The present study indicated that plasma endocannabinoids levels, especially AEA, were associated with IR and AHI in patients with OSA.

## 1. Introduction

Obstructive sleep apnea (OSA) is a condition characterized by partial or complete upper airway obstruction and intermittent hypoxia (IH) occurring repeatedly during sleep. OSA is a major health problem affecting 10% of men and 3% of women in the general population [1].

OSA is closely associated with insulin resistance (IR) and has been shown to be an independent risk factor for incident diabetes [2]. Population studies have shown that OSA was positively related to the degree of IR, and the more severe the OSA, the greater the IR [3]. Furthermore, a lot of studies have proved that OSA per se can cause and aggravate IR, which is independent of obesity and other factors that cause IR [4]. Continuous positive airway pressure (CPAP) can reverse the IR of OSA [5]. Moreover, healthy volunteers in IH condition simulating moderate OSA showed a trend of decreased insulin sensitivity and glucose effectiveness after five hours [6]. Similar results can be observed in the animal model. In a nonobese rodent model of OSA, chronic intermittent hypoxia (CIH) led to IR and impaired glucose tolerance [7].

The endocannabinoid system (ECS) is crucial in the regulation of metabolism and energy homeostasis [8]. It consists of cannabinoid receptors, endocannabinoids, and the enzymes involved in their biosynthesis and degradation and is present both in the brain and peripheral tissues. Endocannabinoids are produced by virtually all cell types; however the most important sources of endocannabinoids are vascular smooth muscle cells, endothelial cells, nerve cells and circulating blood cells [9]. There are many kinds of endocannabinoids, including anandamide (AEA), 2-arachidonoylglycerol (2-AG), N-palmitoylethanolamine (PEA), and N-oleoylethanolamine (OEA). Of these endocannabinoids, AEA and 2-AG are the most important and the most concerned ones. There are two G protein-coupled cannabinoid receptors, CB1 and CB2. CB1 activation is associated with insulin resistance and dyslipidemia. Activation of CB1 results in increased food intake, insulin resistance by inhibiting glucose uptake into skeletal muscle [10]. Accordingly, CB1 blockade or genetic knockout of CB1 results in decreased food intake and body weight, increased insulin sensitivity, and improvements in glucose homeostasis through an increased insulin-mediated glucose transport in skeletal muscles [11, 12], and similar effects were reported in obese subjects treated with CB1 receptor antagonist rimonabant [13]. Circulating endocannabinoids were increased in obese patients, particularly with visceral obesity and in patients with IR [14]. AEA, 2-AG, and OEA plasma concentrations in sleep apnea patients were considerably increased compared to healthy sleepers [15, 16].

Whereas the association between OSA and IR and the relationship between ECS and IR have been clearly documented, it is still unclear whether the ECS plays a role in the process of IR in OSA patients. Therefore, the main purpose of the present study was to examine plasma levels of AEA and 2-AG in OSA patients and its possible relationship with OSA and IR.

## 2. Materials and Methods

### 2.1. Study Subjects

This study was approved by the ethics committee of the First Hospital of Lanzhou University. These participants were recruited in the First Hospital of Lanzhou University and signed an informed consent. 88 consecutive participants who referred to suspected sleep disordered breathing were assessed and 64 who fulfilled the OSA criteria were recruited as OSA group. Others with negative OSA evaluation results were recruited as control group. OSA was diagnosed by doing one overnight polysomnography (PSG) and the data was scored manually using a computerized polysomnogram system (Polywin, USA). Due to stress and other reasons, some subjects' monitoring results were not true for the first time, and another PSG may be arranged, until achieving the desired results. Sleep recording included electroencephalogram (EEG), electrooculogram (EOG), chin electromyogram, airflow, oxygen saturation, respiratory effort, and electrocardiogram (ECG). Apnea was defined as complete cessation of airflow for more than 10 seconds.

Hypopnea was defined as a discrete reduction (two-thirds) of airflow and/or abdominal ribcage movements lasting more than 10 seconds and associated with a decrease of at least 3% in oxygen desaturation. Apnea-hypopnea index (AHI) was defined as the number of apnea or hypopnea events per hour during sleep. OSA was diagnosed when the AHI exceeded the threshold of ≥5 per hour. Patients with confirmed OSA were subsequently grouped according to the disease severity into a mild OSA group (5/h ≤ AHI < 15/h), a moderate OSA group (15/h ≤ AHI < 30/h), and a severe OSA group (AHI ≥ 30/h). Subjects cannot be included in the study if they had been previously diagnosed with OSA, diabetes mellitus, hypertension, cancer, liver disease, kidney disease, autoimmune disorders, infectious diseases, and other endocrine disorders. In addition, we also excluded subjects who received glucocorticoids, antihypertensive, antiobesity, and lipid lowering drugs, and any other medication that may affect glucose, insulin, or endocannabinoids levels. All study subjects will carry out a full medical examination, including collection of medical history and taking a physical examination by qualified physicians.

### 2.2. Baseline Clinical and Biochemical Information

All baseline clinical measurements were performed in the resting state in the morning after PSG. Baseline clinical measurements consisted of age, gender, weight, height, body mass index (BMI), waist circumference, systolic blood pressure (SBP), diastolic blood pressure (DBP), past medical history, and treatment. Then 10 mL venous blood samples were taken for biochemical examination and endocannabinoids measurements. Biochemical information includes blood lipid profile [total cholesterol (TC), low density lipoprotein-cholesterol (LDL-C), high density lipoprotein-cholesterol (HDL-C), and triglycerides (TGs)], fasting blood glucose, and fasting blood insulin. Biochemical analyses were performed at a laboratory in the First Hospital of Lanzhou University (Lanzhou, Western China) with conventional experimental method on fresh samples of blood. Blood glucose was measured in plasma and other indicators were measured in serum. All samples were measured in duplicate.

### 2.3. Measurements of IR

IR was assessed using the homeostasis model of assessment for insulin resistance index (HOMA-IR). It can be calculated using the Oxford University online calculator (http://www.dtu.ox.ac.uk/homacalculator/, accessed June 7, 2015) [17]. HOMA %  *S* represents values of 100% in normal adults when using currently available assays for insulin or C-peptide. HOMA-IR is the reciprocal of HOMA %  *S*. The validity and accuracy of these measurements have been verified and HOME-IR was confirmed with a very high correlation of the glucose clamp test [17].

### 2.4. Measurement of Plasma Endocannabinoids Levels

5 mL of venous blood samples was taken on heparin, immediately centrifuged free of erythrocytes at 4°C, and then kept frozen at −80°C until further analysis. Because 1-arachidonoylglycerol and 2-arachidonoylglycerol rapidly transform into each other during the preanalytical period and the analytical process, statistical analysis included always the sum of 1-arachidonoylglycerol and 2-arachidonoylglycerol (1/2-AG). AEA and 1/2-AG were quantified by liquid chromatography-mass spectrometry (Agilent Technologies Inc., Santa Clara, CA), as previously described [18].

### 2.5. Statistical Analysis

Normal distribution of all variables within each group was confirmed with the Kolmogorov-Smirnov test. Data is presented as mean ± standard deviation (SD). *t*-test was applied for bivariate analyzing and one-way ANOVA and least significant difference (SLD) for multivariate analyzing. Comparisons of frequency variables use the chi square test. Pearson's coefficient was calculated for correlation analysis. Partial correlation analyses were used to test the relationship between two variables while adjusting for potential confounding factors. Multiple regression analyses were performed to identify variables that could be best associated with HOMA-IR in OSA patients. All of the statistical analyses were performed with IBM SPSS 19.0 (SPSS Inc., Chicago, Illinois, USA). All statistical tests were two-sided, with *P* < 0.05 taken to indicate statistical significance.

## 3. Results

### 3.1. Participant Characteristics

Clinical and biochemical characteristics of OSA group and control group were given in Table 1. Two groups were well matched for age, BMI, and male/female proportion. Besides some obvious differences in polysomnogram, OSA group had significantly higher TC, LDL-C, fasting insulin, HOMA-IR, AEA, and 1/2-AG and lower HDL-C compared to control group (all *P* < 0.05). Compared to control group, OSA group also had slightly higher waist circumference, TGs, and fasting glucose levels, but not statistically significant from those in the control group (all *P* > 0.05). Clinical and biochemical characteristics of three subgroups of OSA patients were shown in Table 2. Except for age, sex ratio, waist circumference, TGs, and fasting glucose, other indicators showed statistically significant difference among the subgroups in OSA patients (all *P* < 0.05).

### 3.2. IR in All Groups and the Relationship between IR and AHI in OSA Patients

**Figure 1:**
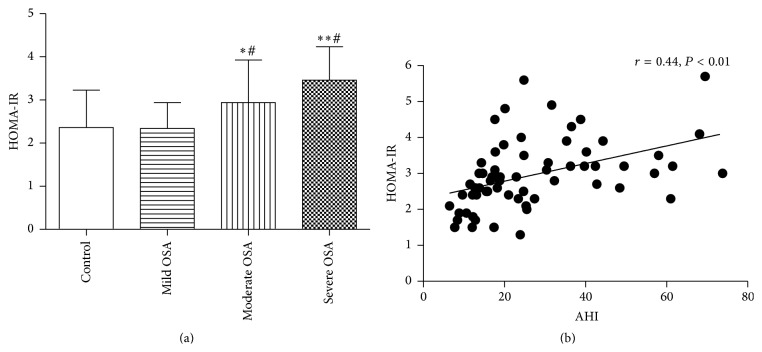
HOMA-IR in four groups (a) and the relationship between HOMA-IR and AHI in OSA patients (b). ^*∗*^ *P* < 0.05 versus mild OSA, ^*∗∗*^ *P* < 0.05 versus moderate OSA, and ^#^ *P* < 0.05 versus control group.

All group's HOMA-IR were shown in Tables 1 and 2 and Figure 1. The severe OSA patients had significant higher HOMA-IR than moderate OSA patients (*P* = 0.04), and moderate OSA patients had significant higher HOMA-IR than mild OSA patients (*P* = 0.02). The mild OSA patients have similar HOMA-IR with control subjects (*P* > 0.05). To investigate associations of IR with AHI, a correlation analysis was performed. As shown in Figure 1(b), there is a significant positive correlation between HOMA-IR and AHI (*r* = 0.44; *P* < 0.01). Partial correlation analysis showed that the significant correlation between HOMA-IR and AHI was not changed until AEA, but not other indicators, was controlled.

### 3.3. The Endocannabinoids in All Groups and the Relationship between Endocannabinoids and AHI in OSA Patients

**Figure 2:**
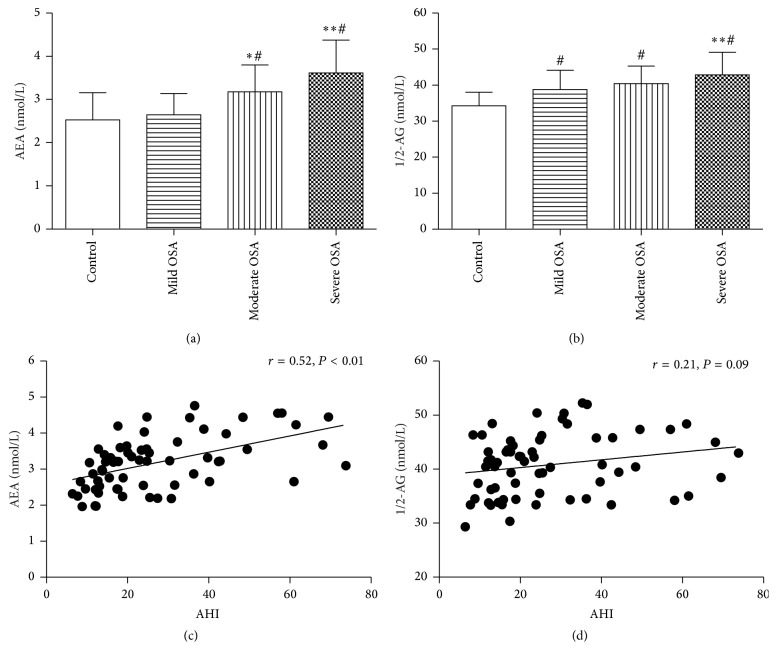
Plasma AEA and 1/2-AG level in all groups (a), (b) the relationship between AEA and AHI (c), 1/2-AG and AHI (d) in OSA patients. ^*∗*^ *P* < 0.05 versus mild OSA, ^*∗∗*^ *P* < 0.05 versus moderate OSA, and ^#^ *P* < 0.05 versus control group.

Plasma concentrations of AEA and 1/2-AG in all groups were shown in Tables 1 and 2, Figures 2(a) and 2(b). AEA and 1/2-AG were significantly increased in severe OSA patients in comparison to mild OSA patients and control (*P* < 0.05). As for AEA, severe OSA patients had higher plasma AEA level than moderate OSA patients (*P* < 0.05) and moderate OSA patients had higher plasma AEA level than mild OSA patients and control subjects (*P* < 0.05), and the latter two had similar AEA (*P* > 0.05). As for 1/2-AG, each of the OSA groups had a higher level of 1/2-AG than control subjects and severe OSA had a high level of 1/2-AG compared with moderate OSA and mild OSA (all *P* < 0.05). Moderate OSA and mild OSA had similar 1/2-AG level (*P* > 0.05). Thus, to further analyze the association of AEA and 1/2-AG with AHI, a correlation analysis was performed between AEA, 1/2-AG, and AHI.  Figure 2(c) showed a significant positive correlation between AEA and AHI (*r* = 0.52; *P* < 0.01). A positive but no significant correlation between 1/2-AG levels and the AHI was shown in Figure 2(d) (*r* = 0.21; *P* = 0.09). Partial correlation analysis showed that plasma AEA level was still significantly correlated with AHI (*r* = 0.36, *P* < 0.01) after adjusting for age, BMI, waist circumference, lipid, and HOMA-IR.

### 3.4. Relationship between Endocannabinoids and IR

**Figure 3:**
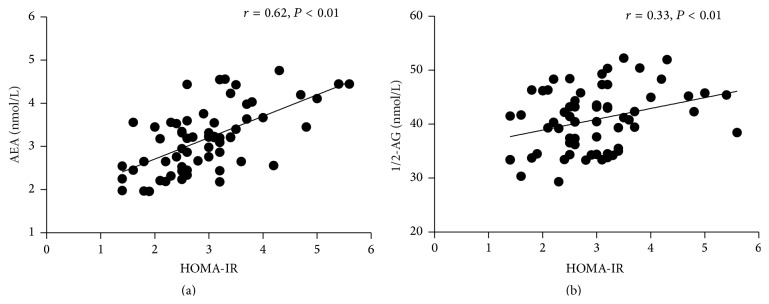
The relationship between AEA and HOMA-IR (a), 1/2-AG and HOMA-IR (b).

To further investigate the relationship between endocannabinoids and IR, an association has been described. In OSA patients, as can be seen in Figures 3(a) and 3(b), there was a significant positive correlation between AEA and HOMA-IR (*r* = 0.62; *P* < 0.01), and there was a significant a positive correlation between 1/2-AG and HOMA-IR (*r* = 0.33; *P* < 0.01). Partial correlation analysis showed that plasma AEA level but not 1/2-AG level was significantly correlated with HOMA-IR (*r* = 0.45; *P* < 0.01) after adjusting for age, BMI, waist circumference, lipid, and AHI. As for control subjects, the correlation between endocannabinoids and IR was not statistically significant (*r* = 0.05, *P* = 0.82 for AEA and *r* = 0.08, *P* = 0.71 for 1/2-AG).

### 3.5. Multiple Regression Analysis to Identify Independent Determinants of HOMA-IR in OSA Patients

To further assess relations between HOMA-IR and other measurements in OSA patients, a multiple stepwise regression analysis was performed with HOMA-IR as a dependent variable; age, gender, BMI, waist circumference, TC, LDL-C, HDL-C, TGs, AHI, mean oxygen saturation, lowest oxygen saturation, oxygen saturation < 90%, AEA, and 2-AG were all included in the regression equation as independent variables. It turned out that HOMA-IR had significant and independent associations with AEA (*R* ^2^ = 0.383) or associations with AEA and TC (*R* ^2^ = 0.439) (Table 3).

## 4. Discussion

Our aim was to examine the association between plasma endocannabinoids and markers of IR in OSA individuals. Results showed that plasma levels of endocannabinoids, especially AEA, were significantly increased in patients with OSA and showed a strong correlation with AHI and HOMA-IR.

IR is the hallmark of type 2 diabetes and can eventually lead to the development of type 2 diabetes. It has been proved that the level of IR is associated with the severity of OSA [19]. Study has shown that AHI increased 1 per hour, HOMA-IR increased by 0.5%, suggesting OSA has a close relationship with IR [20]. Consistent with this piece of information, our study showed that IR measured by HOMA-IR was increased in OSA patients compared to controls group. In OSA group, with the increase in AHI, IR increased too, and there was close correlation between AHI and HOMA-IR regardless of the patient's age, BMI, waist circumference, and lipid. However, the relationship between IR and AHI was weakened after adjustment for AEA. Thus, we speculate that circulating endocannabinoids especially AEA play an important role in OSA induced IR. OSA exhibits complex pathophysiologic mechanisms that may potentially contribute to the development of IR, including autonomic activation, alterations in neuroendocrine function, direct effects of hypoxemia on glucose regulation, and release of proinflammatory cytokines such as interleukin-6 and tumor necrosis factor-alpha [21].

Furthermore, we found higher plasma endocannabinoids (AEA and 1/2-AG) concentrations in patients with sleep apnea compared to BMI matched groups. With the increase in AHI, AEA and 1/2-AG concentrations in the blood gradually increased, and circulating AEA, not 1/2-AG, was positively associated with AHI in the OSA individuals after adjusting for age, BMI, waist circumference, lipid, and HOMA-IR. The results of our study agree with those of Engeli et al. [15]. Yet, the relationship between sleep apnea severity and 1/2-AG was attenuated after adjustment for confounding factors. Therefore, it is unlikely that 1/2-AG and sleep apnea are directly linked with each other. On the whole, this finding may indicate the specific role of endocannabinoids in humans with sleep apnea. The role might include neuroprotection from chronic hypoxic stressors, promotion of wakefulness as a compensatory mechanism in states of sleep deprivation and daytime fatigue [22, 23]. Endocannabinoids may also be involved in the regulation of breathing patterns during sleep and in sleep rhythms [24]. There is no certain explanation for higher level of endocannabinoids in OSA patients. One possible mechanism might be related to decreased endocannabinoids degradation. Study showed previously that excessive endocannabinoids concentrations in obesity are associated with decreased fatty acid amide hydrolase (FAAH) gene expression in adipose tissue [25]. Because obesity and OSA are closely related, we speculate that a similar mechanism could be involved in OSA. In addition, leptin has previously been described to stimulate the FAAH activity [26], and FAAH is the primary hydrolytic enzyme for the AEA and 2-AG, so dysregulation of leptin in OSA group [27] may help explain differences in AEA and 1/2-AG between OSA patients and control individuals. Another possible mechanism might be related to IR itself. Insulin was described as a negative regulator of AEA and the regulatory effect of insulin was diminished by IR [28]. Thus, a gradual loss of the inhibitory effect of insulin may represent a mechanism that explains increased circulating AEA in OSA patients compared to control group. On the other hand, the increased endocannabinoid levels observed in OSA patients may constitute an initial compensatory mechanism to improve insulin function and to overcome IR associated with OSA.

Moreover, another finding of our analysis was that circulating concentrations of the endocannabinoids related to IR in OSA patients but not in no-OSA subjects and AEA can predict IR in OSA patients to a certain extent. It has been previously shown that IR is associated with an increase in the concentrations of endocannabinoids [29]. This was in agreement with our present finding. Our study also showed that when AEA was controlled, the correlation between AHI and IR was not statistically significant, and the correlation between IR and AEA remained statistically significant when AHI was controlled. This might mean that insulin sensitivity of OSA patients is more closely related to plasma AEA level than AHI. Whether and how endocannabinoids affect human insulin sensitivity are unknown. One mechanism is that increased concentrations of endocannabinoids, and subsequent elevation of CB1 receptor activity, are suggested to reduce glucose uptake in skeletal muscle and increase abdominal adiposity and free fatty acid flow from adipose tissue to the liver, thus increasing the risk of IR [30].

The main limitation of our study was that the conclusions we got were mainly based on correlation analysis. It reflected only a superficial relationship between OSA severity, IR, and endocannabinoids, and it is far away from clarifying the exact relationship with each other. Another limitation is that we only measured endocannabinoids in blood samples rather than at the tissue level. What is more, our study did not cover the other parts of ECS, such as cannabinoid receptors and enzymes involved in the biosynthesis and degradation of endocannabinoids. Therefore, it is necessary to carry out further research to elucidate the role of endocannabinoids in the occurrence and development of IR in OSA patients.

In conclusion, the main finding of this study is that the plasma AEA level in patients with OSA increased with severity of the disease and closely correlated with OSA induced IR. The putative IR effect of endocannabinoids in OSA remains to be illuminated. Nevertheless, our analysis may provide new insight into relevant mechanisms in sleep apnea-induced IR, and modulation of endocannabinoids metabolism could be an interesting target for pharmacological treatment of IR of OSA.

## Figures and Tables

**Table 1:** Clinical and biochemical characteristics of participants.

Measurement	Controls (*n* = 24)	OSA (*n* = 64)	*P* value
Age (years)	55.2 ± 7.5	54.6 ± 8.4	0.78
Women/men	9/15	22/42	0.81
BMI (kg/m^2^)	27.7 ± 2.7	27.6 ± 3.4	0.92
Waist circumference (cm)	89.0 ± 3.8	90.6 ± 4.0	0.09
TC (mmol/L)	4.22 ± 0.62	4.98 ± 0.55	<0.01
LDL-C (mmol/L)	2.74 ± 0.42	3.19 ± 0.38	<0.01
HDL-C (mmol/L)	1.24 ± 0.21	1.12 ± 0.19	0.02
TGs (mmol/L)	2.07 ± 0.23	2.18 ± 0.28	0.08
AHI (events/h)	3.15 ± 1.04	27.09 ± 16.95	<0.01
Mean oxygen saturation (%)	95.7 ± 1.0	89.8 ± 3.3	<0.01
Lowest oxygen saturation (%)	87.8 ± 2.5	73.4 ± 8.5	<0.01
Oxygen saturation < 90% (%)	0.40 ± 0.15	23.39 ± 4.67	<0.01
Fasting glucose (mmol/L)	5.22 ± 0.72	5.60 ± 0.95	0.08
Fasting insulin (mlU/L)	18.5 ± 7.2	22.9 ± 7.8	0.02
HOMA-IR	2.37 ± 0.89	2.95 ± 0.92	<0.01
AEA (nmol/L)	2.53 ± 0.63	3.09 ± 0.77	<0.01
1/2-AG (nmol/L)	34.3 ± 7.7	40.8 ± 5.7	<0.01

Data is presented as mean ± SD. Differences were considered significant when *P* < 0.05.

**Table 2:** Clinical and biochemical characteristics of OSA patients.

Measurement	Mild OSA (*n* = 18)	Moderate OSA (*n* = 24)	Severe OSA (*n* = 22)	*P* value
Age (years)	55.3 ± 9.3	52.5 ± 8.3	56.5 ± 7.5	>0.05
Women/men	6/12	8/16	8/12	>0.05
BMI (kg/m^2^)	26.2 ± 2.2	27.2 ± 2.7	29.3 ± 4.6	<0.05
Waist circumference (cm)	90.0 ± 3.9	90.9 ± 3.9	90.9 ± 4.3	>0.05
TC (mmol/L)	4.46 ± 0.60	4.91 ± 0.6	5.35 ± 0.67	<0.05
LDL-C (mmol/L)	2.98 ± 0.41	3.27 ± 0.37	3.36 ± 0.25	<0.05
HDL-C (mmol/L)	1.17 ± 0.10	1.15 ± 0.17	1.04 ± 0.24	<0.05
TGs (mmol/L)	2.15 ± 0.24	2.22 ± 0.30	2.15 ± 0.29	>0.05
AHI (events/h)	11.5 ± 2.4	20.8 ± 3.7	46.7 ± 13.8	<0.01
Mean oxygen saturation (%)	92.4 ± 1.5	89.9 ± 2.6	87.5 ± 3.4	<0.01
Lowest oxygen saturation (%)	80.2 ± 5.6	75.7 ± 5.0	65.4 ± 6.9	<0.01
Oxygen saturation < 90% (%)	20.9 ± 3.9	22.8 ± 3.9	26.0 ± 4.9	<0.01
Fasting glucose (mmol/L)	5.36 ± 0.79	5.57 ± 0.99	5.84 ± 0.99	>0.05
Fasting insulin (mlU/L)	17.7 ± 4.5	22.9 ± 8.4	27.2 ± 6.9	<0.01
HOMA-IR	2.34 ± 0.59	2.94 ± 0.99	3.46 ± 0.77	<0.01
AEA (nmol/L)	2.65 ± 0.49	3.18 ± 0.62	3.61 ± 0.77	<0.01
1/2-AG (nmol/L)	38.8 ± 5.3	40.4 ± 4.9	40.9 ± 6.3	>0.05

Data are presented as mean ± SD. Differences were considered significant when *P* < 0.05.

**Table 3:** Stepwise multiple regression analysis to reveal independent associated factors with HOMA-IR.

Model	Included variables	*β*	*t*	*P*	*r*	*R* ^2^
1	(Constant)		1.240	0.220		0.383
	AEA	0.619	6.199	0.000	0.619	

2	(Constant)		−1.252	0.215		0.439
	AEA	0.574	5.875	0.000	0.564	
	TC	0.241	2.469	0.016	0.237	

## References

[1] Kuo T. B. J., Yuan Z. F., Lin Y. S. (2011). Reactive oxygen species are the cause of the enhanced cardiorespiratory response induced by intermittent hypoxia in conscious rats. *Respiratory Physiology & Neurobiology*.

[2] Botros N., Concato J., Mohsenin V., Selim B., Doctor K., Yaggi H. K. (2009). Obstructive sleep apnea as a risk factor for type 2 diabetes. *The American Journal of Medicine*.

[3] Wang T., Zhou Y. T., Chen X. N., Zhu A. X. (2014). Putative role of ischemic postconditioning in a rat model of limb ischemia and reperfusion: involvement of hypoxia-inducible factor-1*α* expression. *Brazilian Journal of Medical and Biological Research*.

[4] Peppard P. E., Young T., Barnet J. H., Palta M., Hagen E. W., Hla K. M. (2013). Increased prevalence of sleep-disordered breathing in adults. *American Journal of Epidemiology*.

[5] Yang D., Liu Z., Yang H., Luo Q. (2013). Effects of continuous positive airway pressure on glycemic control and insulin resistance in patients with obstructive sleep apnea: a meta-analysis. *Sleep & Breathing*.

[6] Louis M., Punjabi N. M. (2009). Effects of acute intermittent hypoxia on glucose metabolism in awake healthy volunteers. *Journal of Applied Physiology*.

[7] Fu C., Jiang L., Zhu F. (2015). Chronic intermittent hypoxia leads to insulin resistance and impaired glucose tolerance through dysregulation of adipokines in non-obese rats. *Sleep & Breathing*.

[8] Silvestri C., Ligresti A., Di Marzo V. (2011). Peripheral effects of the endocannabinoid system in energy homeostasis: adipose tissue, liver and skeletal muscle. *Reviews in Endocrine & Metabolic Disorders*.

[9] Reinke C., Bevans-Fonti S., Drager L. F., Shin M.-K., Polotsky V. Y. (2011). Effects of different acute hypoxic regimens on tissue oxygen profiles and metabolic outcomes. *Journal of Applied Physiology*.

[10] Song D., Bandsma R. H. J., Xiao C. (2011). Acute cannabinoid receptor type 1 (CB1R) modulation influences insulin sensitivity by an effect outside the central nervous system in mice. *Diabetologia*.

[11] Nam D. H., Lee M. H., Kim J. E. (2012). Blockade of cannabinoid receptor 1 improves insulin resistance, lipid metabolism, and diabetic nephropathy in db/db mice. *Endocrinology*.

[12] Tam J., Godlewski G., Earley B. J. (2014). Role of adiponectin in the metabolic effects of cannabinoid type 1 receptor blockade in mice with diet-induced obesity. *American Journal of Physiology—Endocrinology and Metabolism*.

[13] Bergholm R., Sevastianova K., Santos A. (2013). CB_1_ blockade-induced weight loss over 48 weeks decreases liver fat in proportion to weight loss in humans. *International Journal of Obesity*.

[14] Blüher M., Engeli S., Klöting N. (2006). Dysregulation of the peripheral and adipose tissue endocannabinoid system in human abdominal obesity. *Diabetes*.

[15] Engeli S., Blüher M., Jumpertz R. (2012). Circulating anandamide and blood pressure in patients with obstructive sleep apnea. *Journal of Hypertension*.

[16] Jumpertz R., Wiesner T., Blüher M. (2010). Circulating endocannabinoids and *N*-acyl-ethanolamides in patients with sleep apnea—specific role of oleoylethanolamide. *Experimental and Clinical Endocrinology & Diabetes*.

[17] Goto S., Takahashi R., Radak Z., Sharma R. (2007). Beneficial biochemical outcomes of late-onset dietary restriction in rodents. *Annals of the New York Academy of Sciences*.

[18] Wang L., Liu J., Harvey-White J., Zimmer A., Kunos G. (2003). Endocannabinoid signaling via cannabinoid receptor 1 is involved in ethanol preference and its age-dependent decline in mice. *Proceedings of the National Academy of Sciences of the United States of America*.

[19] Kamata H., Honda S.-I., Maeda S., Chang L., Hirata H., Karin M. (2005). Reactive oxygen species promote TNFalpha-induced death and sustained JNK activation by inhibiting MAP kinase phosphatases. *Cell*.

[20] Yang J., Park Y., Zhang H. (2009). Feed-forward signaling of TNF-alpha and NF-kappaB via IKK-beta pathway contributes to insulin resistance and coronary arteriolar dysfunction in type 2 diabetic mice. *The American Journal of Physiology—Heart and Circulatory Physiology*.

[21] Gao Z., Hwang D., Bataille F. (2002). Serine phosphorylation of insulin receptor substrate 1 by inhibitor *κ*B kinase complex. *The Journal of Biological Chemistry*.

[22] Iiyori N., Alonso L. C., Li J. (2007). Intermittent hypoxia causes insulin resistance in lean mice independent of autonomic activity. *American Journal of Respiratory and Critical Care Medicine*.

[23] Yokoe T., Alonso L. C., Romano L. C. (2008). Intermittent hypoxia reverses the diurnal glucose rhythm and causes pancreatic beta-cell replication in mice. *The Journal of Physiology*.

[24] Leto D., Saltiel A. R. (2012). Regulation of glucose transport by insulin: traffic control of GLUT4. *Nature Reviews Molecular Cell Biology*.

[25] Drager L. F., Li J., Reinke C., Bevans-Fonti S., Jun J. C., Polotsky V. Y. (2011). Intermittent hypoxia exacerbates metabolic effects of diet-induced obesity. *Obesity*.

[26] Morton N. M. (2010). Obesity and corticosteroids: 11beta-hydroxysteroid type 1 as a cause and therapeutic target in metabolic disease. *Molecular and Cellular Endocrinology*.

[27] Pallayova M., Lazurova I., Donic V. (2011). Hypoxic damage to pancreatic beta cells—the hidden link between sleep apnea and diabetes. *Medical Hypotheses*.

[28] Harsch I. A., Schahin S. P., Brückner K. (2004). The effect of continuous positive airway pressure treatment on insulin sensitivity in patients with obstructive sleep apnoea syndrome and type 2 diabetes. *Respiration*.

[29] Walter C., Ferreirós N., Bishay P., Geisslinger G., Tegeder I., Lötsch J. (2013). Exogenous delta^9^-tetrahydrocannabinol influences circulating endogenous cannabinoids in humans. *Journal of Clinical Psychopharmacology*.

[30] Jun J., Polotsky V. Y. (2007). Sleep-disordered breathing and metabolic effects: evidence from animal models. *Sleep Medicine Clinics*.

