# Corrigendum to “Circulating Endocannabinoids and Insulin Resistance in Patients with Obstructive Sleep Apnea”

**DOI:** 10.1155/2018/5080563

**Published:** 2018-08-23

**Authors:** Xiaoya Wang, Qin Yu, Hongmei Yue, Jiabin Zhang, Shuang Zeng, Fenfen Cui

**Affiliations:** ^1^Department of Respiratory Medicine, The First Hospital of Lanzhou University, Lanzhou 730000, China; ^2^The First Clinical Medical College of Lanzhou University, Lanzhou 730000, China

In the article titled “Circulating Endocannabinoids and Insulin Resistance in Patients with Obstructive Sleep Apnea” [1], a grant number was missing. The Acknowledgments section should be updated as follows:

This study was supported by a grant from the Health Bureau of Gansu Province (no. GSWSKY-2014-28) and a grant from the Natural Science and Technology Fund of Gansu province (no. 1506RJZA254).

## References

[1] Wang X., Yu Q., Yue H., Zhang J., Zeng S., Cui F. (2016). Circulating endocannabinoids and insulin resistance in patients with obstructive sleep apnea. *BioMed Research International*.

